# Altered interaction of physiological activity and behavior affects risky decision-making in ADHD

**DOI:** 10.3389/fnhum.2023.1147329

**Published:** 2023-04-20

**Authors:** Eva Halbe, Fabian Kolf, Alina Sophie Heger, Philippa Hüpen, Moritz Bergmann, Behrem Aslan, Ben J. Harrison, Christopher G. Davey, Alexandra Philipsen, Silke Lux

**Affiliations:** ^1^Department of Psychiatry and Psychotherapy, University of Bonn, Bonn, Germany; ^2^Department of Psychiatry, Psychotherapy and Psychosomatics, Faculty of Medicine, RWTH Aachen University, Aachen, Germany; ^3^JARA–Translational Brain Medicine, Aachen, Germany; ^4^Department of Psychiatry, University of Melbourne, Melbourne, VIC, Australia

**Keywords:** attention deficit and hyperactivity disorder, risky decision-making behavior, skin conductance response (SCR), autonomic nervous system, physiological activity, affective functions, emotional arousal, balloon analogue risk task (BART)

## Abstract

**Background:**

Adult attention-deficit/hyperactivity disorder (ADHD) is often associated with risky decision-making behavior. However, current research studies are often limited by the ability to adequately reflect daily behavior in a laboratory setting. Over the lifespan impairments in cognitive functions appear to improve, whereas affective functions become more severe. We assume that risk behavior in ADHD arises predominantly from deficits in affective processes. This study will therefore aim to investigate whether a dysfunction in affective pathways causes an abnormal risky decision-making (DM) behavior in adult ADHD.

**Methods:**

Twenty-eight participants with ADHD and twenty-eight healthy controls completed a battery of questionnaires regarding clinical symptoms, self-assessment of behavior and emotional competence. Furthermore, skin conductance responses were measured during the performance in a modified version of the Balloon Analogue Risk Task. A linear mixed-effects model analysis was used to analyze emotional arousal prior to a decision and after feedback display.

**Results:**

Results showed higher emotional arousal in ADHD participants before decision-making (β = −0.12, SE = 0.05, *t* = −2.63, *p* < 0.001) and after feedback display (β = −0.14, SE = 0.05, *t* = −2.66, *p* = 0.008). Although risky behavior was greater in HC than in ADHD, we found a significant interaction effect of group and anticipatory skin conductance responses regarding the response behavior (β = 107.17, SE = 41.91, *t* = 2.56, *p* = 0.011). *Post hoc* analyses revealed a positive correlation between anticipatory skin conductance responses and reaction time in HC, whereas this correlation was negative in ADHD. Self-assessment results were in line with the objective measurements.

**Conclusion:**

We found altered changes in physiological activity during a risky decision-making task. The results confirm the assumption of an aberrant relationship between bodily response and risky behavior in adult ADHD. However, further research is needed with respect to age and gender when considering physiological activities.

## 1. Introduction

Attention-deficit/hyperactivity disorder (ADHD) is a common psychiatric disorder with a prevalence of 5% worldwide (Polanczyk et al., 2007). ADHD presents with heterogeneous symptomatology characterized by inattention, impulsivity, and hyperactivity. Patients are also significantly affected in several aspects of daily life including work performance, planning, decision-making (DM), and psychosocial interactions (Must et al., 2013; Polanczyk et al., 2014). A meta-analysis on longitudinal studies has shown that up to 50% of diagnosed children still meet partial ADHD-related symptoms by the age of 25 (Faraone et al., 2006). However, across the lifespan the main symptom of hypermotoric behavior shifts to an inner restlessness, inattention, and emotional dysregulation (Gibbins et al., 2010; Francx et al., 2015). Furthermore, it is noteworthy that adult ADHD has an increasing tendency to engage in risky behavior that derives from inadequate DM. Those heightened risk taking behaviors are seen particularly in situations of risky driving, risky sexual behavior and pathological gambling (Flory et al., 2006; Thompson et al., 2007; Faregh and Derevensky, 2011).

Investigating risky behavior in the context of disadvantageous decisions is challenging in an experimental setting and study results are often inconsistent and do not reflect the daily behavioral deficits of ADHD (Mowinckel et al., 2015; Dekkers et al., 2016). It is often not clear which neuropsychological functions are responsible during the performance of risk-taking paradigms. Furthermore, the wide variety of methodologies makes it difficult to compare different studies with each other. However, according to the Dual Pathway Model (DPM) it is known that two different signaling pathways are involved in the development of behavioral actions: including cognitive-analytic functions (*cold functions*) and the intuitive-affective functions (*hot functions*) (Sonuga-Barke, 2002). The most common decision-making paradigms; IOWA Gambling Task (IGT), Columbia Card Task (CCT-cold), and Game of Dice Task (GDT) involve cognitive-analytic functions, whereas the Balloon Analogue Risk Task (BART) and Colorado Balloon Game (CBG) can be associated with more affective-emotional driven behavior (Lejuez et al., 2002; Brand et al., 2007; Figner et al., 2009; Crowley et al., 2010; Mäntylä et al., 2012).

Due to the heterogeneity in study results regarding risky DM in ADHD, as well as the lack of focus on *hot function* driven behavioral impairments, further research on affective functioning is needed. Additionally, based on the prefrontal recovery hypothesis (Halperin and Schulz, 2006), it has been shown that developmental improvements in cognitive control functions may favor a reduction in behavioral disturbances in adult ADHD (Groen et al., 2013; Mowinckel et al., 2015). Conversely, significant increases in impaired emotional competence can be observed and are known to negatively affect the patients’ socio-emotional life (Shushakova et al., 2018; Materna et al., 2019; Beheshti et al., 2020). Moreover, these emotional difficulties are also a risk factor for the development of affective comorbidities, such as major depressive disorder (Bresner et al., 2009). It can thus be assumed that impairments of *cold functions* in adulthood diminish, whereas dysfunctions in emotional control and regulation become more prevalent (Shushakova et al., 2018; Materna et al., 2019; Beheshti et al., 2020; Mayer et al., 2021). This leads to the assumption that heightened risk-taking in adult ADHD that arises from deficits in DM ability may result from an impairment in *hot functions*.

Referring to the DPM, hot functions describe particular behavior that arise quickly and intuitively (Sonuga-Barke, 2002; Mäntylä et al., 2012; Shoham et al., 2016), in which perceived stimuli are transmitted directly from the sensory system to the amygdala (Damasio et al., 2000). Due to this activation of the limbic system, the autonomic nervous system is stimulated and induces physiological responses such as modulations in heart rate, sweating, breathing and eye blinking (Figner and Murphy, 2011; Bellato et al., 2020). These physiological changes, in turn, are perceived by the body in the form of a feedback loop which can then elicit a behavioral action (Christopoulos et al., 2019). Thus, the signaling pathway of hot functions is composed of an interconnection of perception of the stimulus, activation of the autonomic nervous system and physiological changes. Although these processes are not consciously experienced, this interconnection to a certain stimulus can be *learned* and stored as somatic markers in the brain, according to prominent theories (Damasio, 1996). Thus, the somatic response can also act as an early-warning-system that guides subconscious decisions from the learned connection of the stimulus and body reaction.

A promising measurement technique to detect somatic marker functioning and anticipatory physiologically changes, is the recording of skin conductance responses (SCR) (Starcke et al., 2009; Wright and Rakow, 2017; Christopoulos et al., 2019). When emotional arousal occurs the electrical property of the skin changes due to the increased sweat secretion. This can be detected by applying an external direct current with constant voltage to the skin (Boucsein, 2012; Uddin et al., 2017). It is important to determine at which timepoint during a DM process a change in skin conductance occurs. In the present study changes that follow a decision are described as *reactive* SCR (rSCR) whereas changes that precede a decision are described as *anticipatory* SCR (aSCR). Although SCRs represent emotional arousal, it cannot be clearly assessed whether an rSCR follows a positive or negative feedback (Figner and Murphy, 2011). It has only be shown that highly evaluated feedback (either being high rewards or high punishments) were associated with increased SCR amplitudes (Wilkes et al., 2010; Lole et al., 2012). In addition, risky behavior was found to decrease when rSCR was already elevated in the previous trial (Masunami et al., 2009; Wright and Rakow, 2017). Regarding aSCRs, it is well documented that disadvantageous DM behavior is associated with higher amplitudes (Garon et al., 2006; Starcke et al., 2009; Dawson et al., 2011; Wright and Rakow, 2017). Thus, it can be shown that when a high risk condition is present, an increased amplitude in aSCR represents an implicit perception of risk (Guillaume et al., 2009; Rogalsky et al., 2012; Wright and Rakow, 2017; Agren et al., 2019).

There are only a few studies which have investigated the relationship between electrodermal activity and risky DM behavior in adult ADHD. However, results indicated an altered psychophysiological activity in the context of behavioral impairment in adult ADHD (Lazzaro et al., 1999; Lackschewitz et al., 2008; Matthies et al., 2012; Ziegler et al., 2016). Furthermore, a recent systematic review investigating autonomic nervous system function in ADHD identified altered activity patterns at rest but also during task performance. Results were not consistent and showed both hypoactive and hyperactive patterns in ADHD compared to healthy controls (HC) (Bellato et al., 2020). To date, however, much of the research in this area has mainly focused on abnormalities in rSCR regarding feedback sensitivity. In these studies, results indicated greater risky DM in conditions of punishment (DeVito et al., 2008; Masunami et al., 2009), whereas rSCRs have been shown to decrease in response to an error feedback or the omission of rewards (Iaboni et al., 1997; O’Connell et al., 2004). In contrast, hyperactivity of the rSCR is partially observed after rewarding feedback (Masunami et al., 2009; Bellato et al., 2020). However, to the best of our knowledge, no study so far has investigated somatic responses as an anticipatory correlate that guides a quick and intuitive behavior in adult ADHD. Assuming dysfunctional affective processes in ADHD, it can therefore be suggested that (1) alterations of aSCR and rSCR do not occur during risky DM; or (2) increases in aSCRs are not linked to advantageous behavioral actions.

The aim of the present study was to investigate whether a dysfunction in affective pathways is associated with an altered risky DM behavior in adult ADHD. To address this, we analyzed SCRs as well as the relationship between aSCRs and task-behavior. Therefore, we used a modified version of the Balloon Analogue Risk Task (BART) to demand hot function-guided risky decision behavior (Hüpen et al., 2019). The BART has been demonstrated to induce naturalistic risky DM (Lejuez et al., 2002). Moreover, the modified version was designed to trigger emotional arousal and intuitive guided behavior, and to investigate DM depending on the reward magnitude (Hüpen et al., 2019, 2020). Additionally, questionnaires were used to investigate self-assessment of risky behavior and emotional competence.

## 2. Materials and methods

### 2.1. Participants

Fifty-nine participants [*n* = 30 HC and *n* = 29 patients with ADHD] were recruited for the current study and met the following inclusion criteria: aged between 18 and 60 years, fluent German language skills, no neurological diseases, no depressive disorder, no borderline personality disorder, or other psychiatric disorder with psychosis. The patient group was recruited from the outpatient clinic of the Department of Psychiatry and Psychotherapy of the University Hospital Bonn and met the full DSM-V criteria (APA, 2013). Participants ceased taking ADHD-specific medications 24 h prior to the start of the experiment which was additionally objectified by oral questioning on the study day. HCs were recruited via public advertisement on the Internet and flyers. Psychiatric symptoms and comorbidities were assessed by a brief diagnostic interview (Mini-DIPS; Margraf and Schneider, 1994), the Beck Depression Inventory-II (BDI-II; Beck et al., 1996) and the Borderline Symptom List-95 (BSL-95; Bohus et al., 2001). ADHD-related psychopathology was quantified by the Conners Adult Rating Scale (CAARS; Conners et al., 1999) and the validated short version of the Wender Utah Rating Scale (WURS-k; Retz-Junginger et al., 2002). Following data collection, three participants (*n* = 2 HC, *n* = 1 ADHD) were excluded from the subsequent analyses due to missing measurement data, acute suicidality, or extreme outlier values. The study was approved by the Ethics Committee of the Medical Faculty of the University of Bonn (122/21) and all participants gave oral and written informed consent.

### 2.2. Materials

#### 2.2.1. Risky decision-making paradigm: Balloon Analogue Risk Task

In the present study a modified version was used which is intended to measure hot function-guided risky DM behavior (Hüpen et al., 2019). Participants are presented with a dynamically growing balloon on a screen for a duration of 5,000 ms. The increasing size of the balloon coincides with an increasing amount of money and a greater risk of the balloon exploding. Participants are asked to press a response button at a self-determined timepoint in order to gain as much money as possible. As the inflation duration is the same on every trial the balloon explosion is not visually presented to the participants. Following this time interval, a fixation cross (250 ms) is shown followed by the feedback display for 2,500 ms. In case of positive feedback, the amount of the collected money is presented. For negative feedback, the participant will be presented with a burst balloon and “0.00 Euros were won” if the determined timepoint was after the explosion point of the balloon. The total amount (sum of trials so far) appears additionally with every feedback. Each trial is also assigned with a certain gain condition (high or low reward). For this, it is shown by color whether the maximum potential trial gain is a high or low condition. The participant is instructed about the linear relationship of the money increase and explosion probability as well as the reward conditions. In total, every participant perform 60 trials (30 trials per each reward condition). For further information, see Henn et al. (2023).

#### 2.2.2. Self-assessment of risk behavior and emotional competence

To investigate self-assessment of risk perception and behavior (Domain Specific Risk Taking; DOSPERT), as well as emotional competence (“Emotionale Kompetenz Fragebogen”; EKF) two self-report questionnaires were used. A subsequent comparison and analysis with the actual behavior in the DM task provides a further insight into the awareness of the participants’ own behavior. The German validated version of the DOSPERT includes 40 items, representing daily situations that are assigned to one of the following subdomains: Investment, Gambling, Health, Recreational, Ethical and Social (Weber et al., 2002). Using a Likert-Scale (1–5), participants should specify the probability of engagement, the estimated risk and the personal benefit of every item. The EKF includes 62 items on self-assessment of emotional-competence-demanding events divided into four categories: Recognizing own feelings, recognizing emotions of others, regulation and control of own feelings and emotional expressivity (Rindermann, 2009). Ratings were scored using a Likert-Scale (1–5). For the analyses, we used the total score of the emotional competence and the total score of the propensity of risk engagement.

#### 2.2.3. SCR data acquisition and preprocessing

Skin conductance was recorded using a Biopac MP150 system (Biopac Systems Inc., Goleta, CA, United States). Recordings were taken at 5,000 Hz and a direct current excitation of 0.5 V. Two disposable snap (Ag-AgCl) electrodes (11 mm diameter), prepared with a 0.5% saline paste in a neutral base (0.05 molar NaCl) were attached to the thenar and hypothenar eminence of the non-dominant hand. Signals of the skin conductance activity were transmitted via the wireless PPG/EDA BioNomadix Transmitter to the software AcqKnowledge (acquisition and analysis program). The recordings were synchronized with the BART sequence via digital input ports sent by the Presentation^®^ software of neurobehavioral systems. Trigger for the reward condition (high/low), feedback display (gain/loss), response timepoint as well as start-/endpoint of the trial were transformed. To do so, a transition latency was set for 2 ms, and an additional low pass filtering of 1 Hz was applied. Further preprocessing was performed with Ledalab toolbox (V.3.4.8) of Matlab, including smoothing (Gaussian method) and a downsampling to 20 Hz. Using a continuous decomposition analysis (CDA) relevant phasic skin conductance responses (SCR) were extracted from the signal tracks (Benedek and Kaernbach, 2010). The integral of the skin conductance responses (ISCR) was used as a measure for the analyses. As the short peaks in activity represents the phasic driver, one response window was defined 1–6 s with condition display (at the beginning of each trial) and one 1–3 s with feedback display (at the end of each trial). For peak detection, a minimum amplitude criterion of 0.05 μS was used.

### 2.3. Research design

**FIGURE 1:**
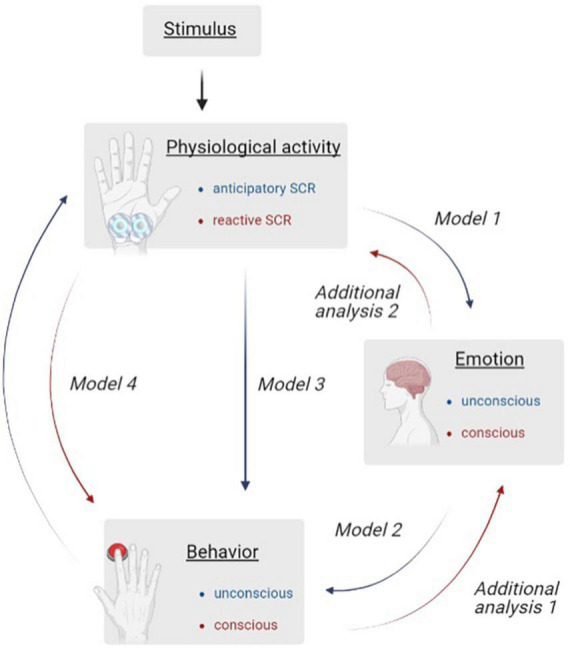
Illustration of the sequential analyses investigating unconscious (blue) and conscious (red) processes during risky decision making. To visualize the process to be investigated, six analyses are shown and assigned via arrow description (Model 1–4; Additional Analyses 1 and 2). The measurement variables belonging to the analysis are highlighted in the boxes “Physiological Activity,” “Emotion,” and “Behavior.”

In order to investigate the research questions on the basis of the materials used, different consecutive analyses are used (see Figure 1). Unconscious (green pathway) and conscious (red pathway) processes from stimulus onset to decision making were examined. As the appearance of the balloon (stimulus) provokes a change in the aSCR that modifies the representing behavior via RT, it should be investigated whether differences occur in the physiological activity (model 1), the behavior (model 2) or whether the behavior is also influenced by the aSCR (model 3). Since feedback also has an impact on hot function guided DM, we also investigated whether differences in rSCR are present (model 4). Two additional analyses should provide information on whether the participants’ own behavior is perceived (additional analysis 1) and how their own affective functions are assessed (additional analysis 2).

### 2.4. Statistical analysis

In order to investigate behavioral and psychophysiological differences between ADHD and HC, we used linear mixed effects model containing interaction terms of the fixed effects and random intercepts for participants and trials. Group (ADHD; HC) and reward condition (high; low) were included as fixed effects in the models. A “Non-responder” was not excluded from analyses as the lack of SCR might represent an actual psychophysiological activity. All models were fitted using the R (R Core Team, 2014) package lme4 (Bates et al., 2015). For *post hoc* comparisons, we used the emmeans package (Lenth, 2016) to account for means and corrected *p*-values. Additional analyses were performed for the evaluation of the two questionnaires (DOSPERT; EKF). Therefore, we used univariate Analysis of Variance (ANOVA) with total scores of each questionnaire being the dependent variable and group (ADHD; HC) representing the independent variable.

## 3. Results

### 3.1. Demographics

There were no significant differences between HC and ADHD in age, gender distribution, verbal intelligence (as assessed by the WST; Metzler and Schmidt, 1992) and years of education (see Table 1). In terms of the clinical screening and the psychiatric symptoms, ADHD had greater levels of self-reported ADHD-related symptoms (Mann–Whitney-*U* = 769.5, *n*1 = *n*2 = 28, *p* < 0.001 two-tailed), depression (Mann–Whitney-*U* = 535, *n*1 = *n*2 = 28, *p* = 0.018 two-tailed) and BPD-related symptoms (Mann–Whitney-*U* = 584.5, *n*1 = *n*2 = 28, *p* = 0.002 two-tailed).

**TABLE 1:** Demographic and clinical characteristics of patients with ADHD and healthy controls (HCs).

		Median		Mann–Whitney-U-test	
**Parameter**		**HC (*n* = 28)**	**ADHD (*n* = 28)**	***U***	***p***
Age (years)		27.0	30.5	431.5	0.516
Verbal IQ (WST)		33.0	32.0	376.5	0.798
Education (years)		18.0	16.0	287	0.178
CAARS	Hyperactivity	8.5	24.5	734.5	<0.001
	Inattention	8.0	25.0	720.5	<0.001
	Impulsivity	6.0	19.5	754.5	<0.001
	Self-conception	4.5	11.5	633.5	<0.001
WURS-k		12.0	40.0	748	<0.001
BDI		2.0	4.5	535	0.018
BSL		2.0	12.0	584.5	0.002
		**Frequency**		**Chi-squared-test**	
Gender (m/f)		9/19	16/12		χ^2^ = 3.54

ADHD, attention-deficit-/hyperactivity disorder; HC, healthy controls; WST, Wortschatztest; CAARS, Conners Adult Rating Scale; WURS-k, Wender Utah Rating Scale; BDI, beck depression inventory; BSL, borderline symptom list; *m* = male; *f* = female.

### 3.2. Unconscious pathway (blue)

**FIGURE 2:**
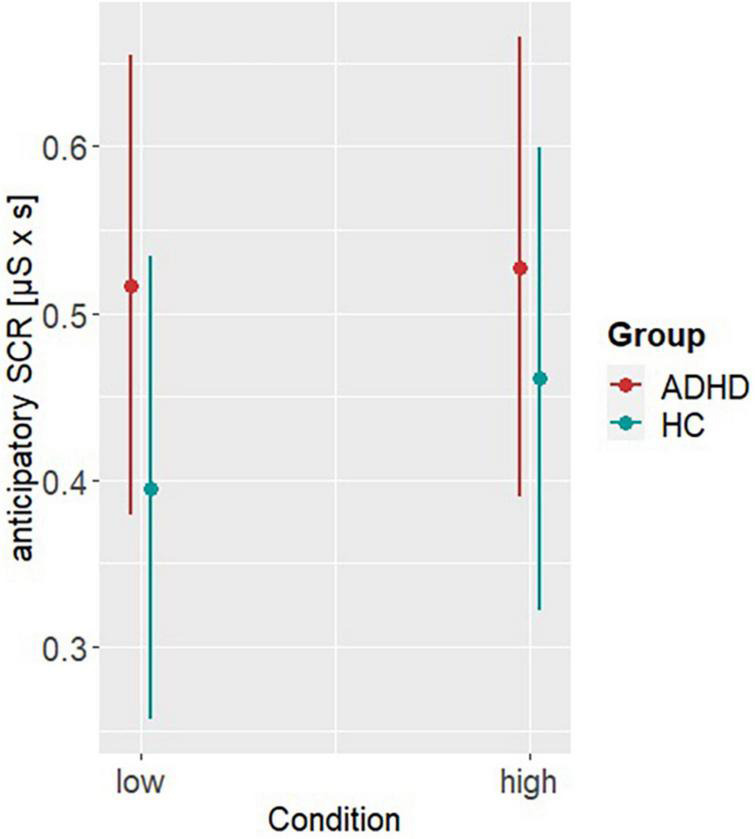
*Post hoc* results of model 1. Representing the anticipatory skin conductance responses (aSCRs) per group (ADHD, HC) and reward condition (low, high).

**FIGURE 3:**
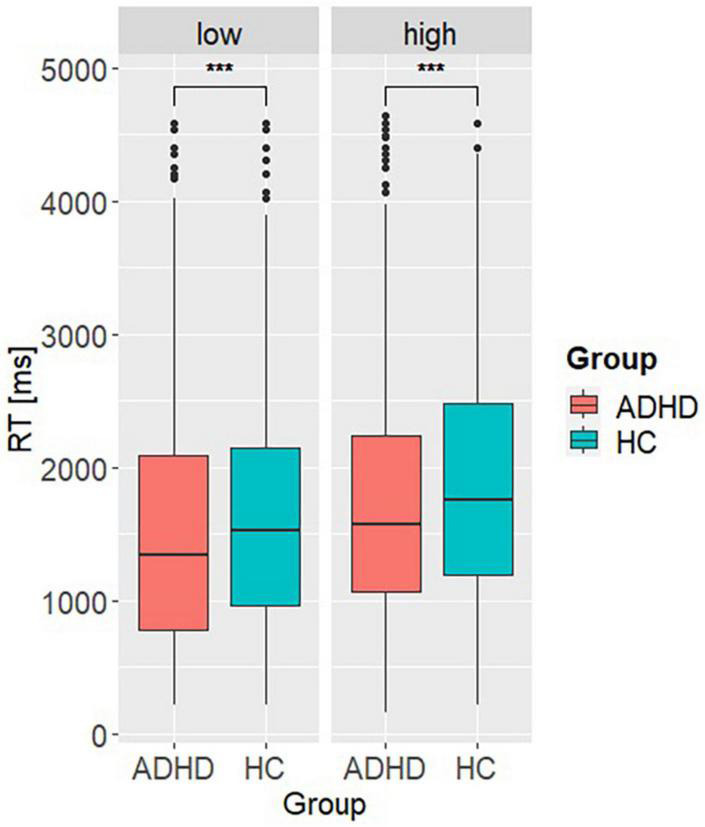
*Post hoc* results of model 2. Representing the reaction time (RT) per group (ADHD, HC) and reward condition (low vs. high). ***p < 0.001.

Model 1 investigating differences in the aSCRs, revealed a significant main effect of group (β = −0.12, SE = 0.05, *t* = −2.63, *p* < 0.001). *Post hoc* analysis showed higher aSCR in ADHD compared to HC (M_Difference_,_ADHD–HC_ = 0.09, SE = 0.03, *t* = 2.79, *p* = 0.005), indicating higher emotional arousal preceding a decision (see Figure 2). Model 2 accounting for the behavioral differences on basis of the RT, revealed a significant main effect of group (β = 219.51, SE = 39.88, *t* = 5.5, *p* < 0.001) and reward condition (β = 215.36, SE = 38.36, *t* = 5.61, *p* < 0.001). *Post hoc* analyses showed higher RTs in HC (M_Difference_,_ADHD–HC_ = −222, SE = 29.3, *t* = −7.59, *p* < 0.001) and under high reward condition (M_Difference_,_low–high_ = −218, SE = 27.2, *t* = −8.02, *p* < 0.001), indicating greater risky DM but also a dependence of behavior on the level of reward in both groups (see Figure 3).

**FIGURE 4:**
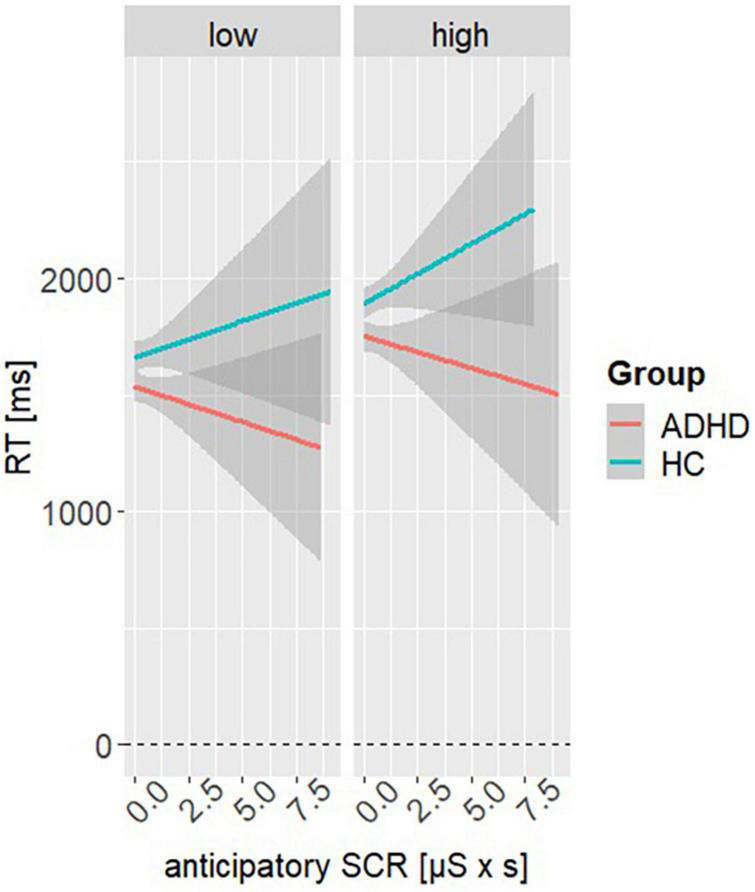
Interaction effect of model 3. Representing the simple slopes for the interaction of anticipatory skin conductance responses (aSCRs) at the factor variables reward condition (high, low) and group (ADHD, HC).

An additional model was used to investigate whether RT is influenced by changes in aSCR. Here, model 3 revealed a significant main effect of aSCR (β = −55.37, SE = 28.03, *t* = −1.98, *p* = 0.048), reward condition (β = 208.91, SE = 43.65, *t* = 4.79, *p* < 0.001) and group (β = 162, SE = 44.38, *t* = 3.66, *p* < 0.001). Furthermore, a significant interaction effect of group and aSCR was found (β = 107.17, SE = 41.91, *t* = 2.56, *p* = 0.011). *Post hoc* analyses showed that RT of HC and ADHD seem to diverge from each other as the aSCR increases, with relationship of aSCR and RT tend to be positive in HC and being negative in ADHD (M_Difference_,_ADHD–HC_ = −222, SE = 29.4, *t* = −7.57, *p* < 0.001). Results indicate risky DM is associated with emotional arousal in HC but not in ADHD (see Figure 4). Please see Table 2 for all other parameter estimates.

**TABLE 2:** Parameter estimates of the linear mixed effects model analyses.

Model		*b*	SE	*t*	CI 95%	*p*
Model 1	Intercept	0.52	0.07	7.34	[0.38, 0.65]	<0.001
	Group	-0.12	0.05	-2.63	[−0.21, −0.03]	<0.001
	Condition	0.01	0.04	0.25	[−0.08, 0.1]	0.8
	Group × condition	0.05	0.06	0.86	[−0.07, 0.18]	0.39
Model 2	Intercept	1327.45	81.08	16.37	[1168.5, 1486.4]	<0.001
	Group	219.51	39.88	5.5	[141.32, 297.71]	<0.001
	Condition	215.36	38.36	5.61	[140.15, 111.31]	<0.001
	Group × condition	4.85	54.3	0.09	[−101.61, 111.31]	0.929
Model 3	Intercept	1438.17	79.33	18.13	[1282.63, 1593.72]	<0.001
	aSCR	-55.37	28.03	-1.98	[−110.32, −0.41]	0.048
	Condition	208.91	43.65	4.79	[123.33, 294.5]	<0.001
	Group	162.63	44.38	3.66	[75.62, 249.65]	<0.001
	aSCR × condition	13.55	39.38	0.34	[−63.66, 90.77]	0.73
	aSCR × group	107.17	41.91	2.56	[24.99, 189.34]	0.011
	Condition × group	18.5	61.27	0.3	[−101.63, 138.63]	0.76
	aSCR × condition × group	0.52	58.14	0.009	[−113.48, 114.51]	0.99
Model 4	Intercept	0.51	0.06	7.86	[0.38, 0.63]	<0.001
	Group	-0.14	0.05	-2.66	[−0.24, −0.04]	0.008
	Feedback	-0.11	0.05	0.05	[−0.21, −0.02]	0.02
	Group × feedback	0.08	0.07	0.07	[−0.05, 0.22]	0.23

Linear mixed-effects model with group (ADHD, HC) as fixed factor in every model. Reward condition (high, low) was additionally included as fixed factor in model 1–3. Feedback (gain, loss) was additionally included as fixed factor in model 4. Anticipatory skin conductance response (aSCR) was additionally included as fixed factor in model 3. Dependent variables were aSCR in model 1, mean reaction time (RT) in model 2 and 3 and reactive skin conductance response (rSCR) in model 4. CI, confidence interval; SE, standard error.

### 3.3. Conscious pathway (red)

**FIGURE 5:**
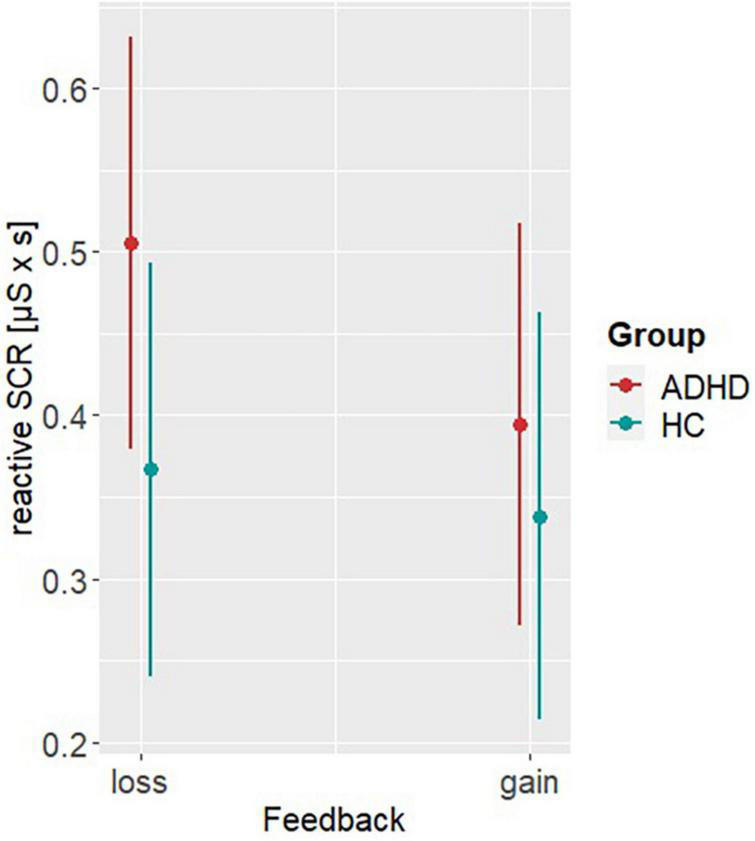
*Post hoc* results of model 4. Representing the reactive skin conductance responses (rSCRs) per group (ADHD, HC) and reward condition (low, high).

Model 4 investigating differences in the rSCRs, revealed significant main effects of group (β = −0.14, SE = 0.05, *t* = −2.66, *p* = 0.008) and feedback (β = −0.11, SE = 0.05, *t* = −2.32, *p* = 0.02). *Post hoc* analyses showed higher rSCRs in ADHD (M_Difference_,_ADHD–HC_ = 0.098, SE = 0.04, *t* = 2.7, *p* = 0.007) and after loss display (M_Difference_,_loss–gain_ = 0.07, SE = 0.03, *t* = 2.03, *p* = 0.042), indicating higher emotional arousal in loss trials with this being more pronounced in ADHD (see Figure 5). Please see Table 2 for all other parameter estimates.

**FIGURE 6:**
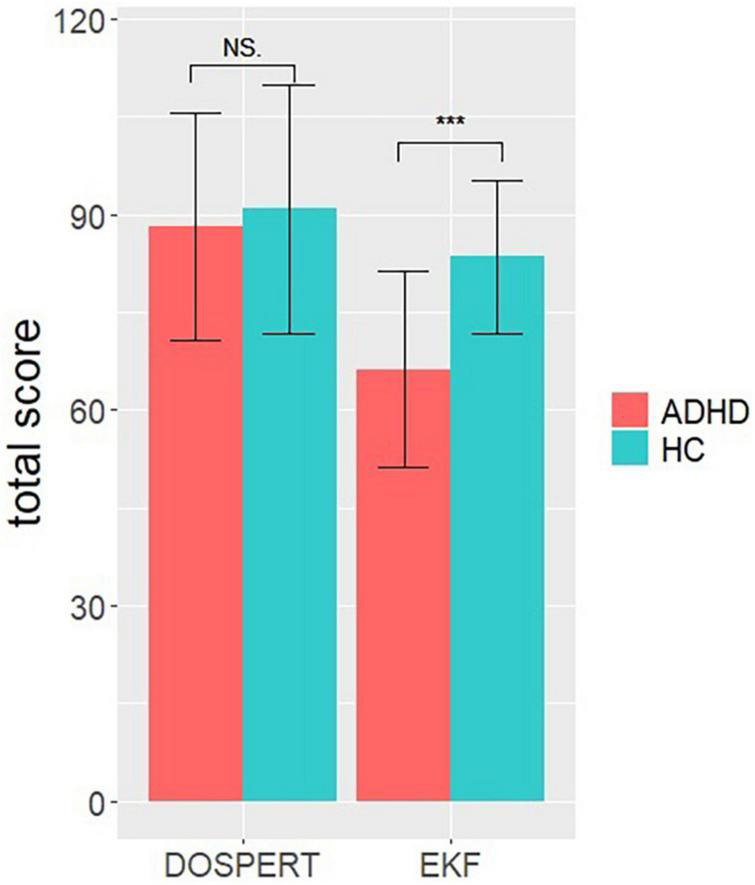
Univariate ANOVA results. Representing the total scores in the questionnaires Domain Specific Risk Taking (DOSPERT) and “Emotionale Kompetenz Fragebogen” (EKF) per group (ADHD, HC). Error bars represent standard errors of the means. NS *p* > 0.05, ***p < 0.001.

The univariate ANOVA for the total score of propensity of risk engagement (additional analysis 1) did not reveal a significant difference of group [*F*_(1_,_54)_ = 0.285, *p* = 0.6, η2 = 0.005]. Results indicate the same propensity of risk engagement in ADHD and HC. The ANOVA for the self-assessment of emotional competence (additional analysis 2) revealed a significant difference between ADHD and HC [*F*_(1_,_54)_ = 23.1, *p* < 0.001, partial η2 = 0.3], indicating a higher emotional competence in HC (see Figure 6).

## 4. Discussion

The present study investigated whether there is a potential causal relationship of affective pathways and abnormal risky DM behavior in adult ADHD. Therefore, different phases of hot function-guided DM were analyzed and compared between adult ADHD and HC on the basis of behavior, physiological activity, and self-assessment.

Results showed higher emotional arousal in ADHD indicated by elevated SCRs preceding a DM. These findings are consistent with the observation of hyperactivity of the autonomic nervous system being increasingly associated with motoric hyperactivity and impulsive behavior in ADHD. These results were suggested to reflect an over-activation of autonomic functions (Wilbertz et al., 2012). It is discussed whether hyperactivity of somatic functions in ADHD serves to induce a stimulating environment in order to promote a certain stability of vigilance (Geissler et al., 2014). In addition, these hyperarousal activities can also be related to a reduced ability of downregulating excessive arousal. However, other findings showed that during the performance of monotonous tasks, quick exhaustion may occur, which is then characterized by hypoactivity of bodily responses (Kuntsi and Klein, 2012; Geissler et al., 2014). It can therefore be assumed that, depending on the demands and excitement of the task; emotional arousal can be either hyper-/or hypo-threshold.

Regarding the available effect of somatic markers at this point, high aSCRs would be expected to be associated with high risk behaviors, as the somatic response will increase with increased risk engagement in order to avoid disadvantageous behavior (Garon et al., 2006; Starcke et al., 2009; Dawson et al., 2011; Wright and Rakow, 2017). However, in the present study risky behavior could not be detected in ADHD. Instead, HCs with overall higher RTs, showed significantly greater risky DM than ADHD. At this point, it can be questioned whether the modified BART accurately reflects daily risky DM and whether it is a valid measurement under laboratory conditions. Moreover, the BART primarily examines risk engagement in the context of financial behaviors and therefore cannot represent all domains of potential daily risk taking behaviors. In addition, recent meta-analyses also highlight the fact that behavioral results of many studies represent rather a suboptimal DM behavior than a risk-seeking DM behavior in ADHD (Dekkers et al., 2021; Roberts et al., 2021). In this context, the underlying mechanism regarding the disentanglement of disadvantageous decisions and risk-seeking decisions reflect an additional important aspect in the field of DM and need to be considered in further studies (Dekkers et al., 2021). On the other hand, the behavior shown could also reflect an action that demonstrates shorter RT according to the Intolerance of Uncertainty that was found to be a transdiagnostic construct in psychiatric disorder (Gramszlo et al., 2018). Subsequently, not the risky DM behavior itself is an interesting outcome, but rather the relationship and interconnection of bodily response and DM.

Thus, in order to investigate how the somatic response influences behavior we analyzed the relation of aSCRs and RT. Results showed that risk engagement in HC coincides with increased aSCR, representing the correlation of disadvantageous behavior and higher SCRs (Garon et al., 2006; Starcke et al., 2009; Dawson et al., 2011; Wright and Rakow, 2017). In turn, increased risk engagement in ADHD was associated with lower aSCR. This negative correlation of RT and aSCR in ADHD confirms the hypothesis about an altered relation between affective state and behavior. However, the strongest affective responses were observed at low RTs. Thus, it is shown that as risk-engagement increases, affectivity decreases, however quick responses tend to elicit the greatest arousal. This suggests that the physical response shown may be related to additional factors than risk. Therefore, it can be assumed that in adult ADHD, there is a missing interconnection between bodily response and behavior regarding risky DM. Nevertheless, further emotionally arousing functions involved in this BART need to be examined.

To understand further aspects of hot function-guided DM, SCRs in response to feedback were also investigated. In this context, the somatic response reflects the evaluation and emotional arousal toward feedback. Results of the rSCRs indicate that feedback is evaluated more emotionally arousing for ADHD than to HC. We could also identify that the feedback of loss is more arousing than gain across both groups. The high rSCRs are also consistent with findings of previous studies on reward sensitivity in ADHD. It was shown that hyperarousal during feedback is associated with weaker inhibitory abilities (Iaboni et al., 1997; Masunami et al., 2009). Furthermore, also in HC it could already be shown that gains produce higher physiological activity (Lole et al., 2012). The impact of feedback, whether it appears as a gain or a loss, can moreover encourage behavioral adaptation. In their prospect theory, Kahneman and Tversky (1979) describe higher emotional arousal and motivation toward loss outcomes. This motivation is accompanied by a decreased propensity to risky DM and is particularly evident in mixed gain/loss prospects. However, it could also be shown that norepinephrine plays a significant role in risk appraisal and propensity (Trepel et al., 2005). Accordingly, it was found that a central norepinephrine blockade decreases the sensitivity to risk-taking significantly (Rogers et al., 2004). Relating this to the underactivity of norepinephrine in ADHD, it seems plausible that reduced risk-taking is caused by the norepinephrine deficiency. Reflecting the elevated rSCRs and the reduced risky DM behavior it can be assumed that the autonomic signal might not get properly transferred to the central nervous system (Critchley and Garfinkel, 2018).

Risk engagement was also investigated by self-assessment using the domain of probability of risk engagement of the DOSPERT questionnaire. Results showed no differences in the self-awareness of risky DM behavior. Contrary to the postulated daily behavior in ADHD, self-assessment does not seem to be perceived as an altered behavior. However, it should be noted that in the current study only the subdomain “probability of risk engagement” was used for a group comparison. Thus, a general evaluation of risky DM based on the DOSPERT results is limited. As questionnaires mostly comprise a subjective and consciously driven self-assessment, it can be assumed that on a conscious level, there are smaller differences in the risk-engagement between HC and ADHD. Consequently, it can be assumed that more unconscious, emotional-motivational driven processes, thus control the increased risky DM behavior in ADHD that is postulated in daily life. This is further supported by the self-assessment of emotional competence using the EKF questionnaire. Results indicated an impaired ability of emotional regulation, perception, understanding, and expression in ADHD that are important requirements for a proper hot function-guided decision (Sonuga-Barke, 2002). Moreover, risky DM is not only guided by previous experience of loss and gain, but also by the potential reward amount. Different studies have shown that with increasing magnitude of reward the risk avoidance also increases (Kahneman, 2003; Van Leijenhorst et al., 2010; Christopoulos et al., 2019; Hüpen et al., 2019). However, the present study indicates greater risk-engagement under high reward conditions, demonstrated by longer RTs, but not by changes in skin conductance. There was, however, no group difference and both ADHD and HC showed greater risky behavior under high reward conditions. Similar results were also shown in studies on ADHD and on Borderline Personality Disorder, arguing that the range between reward conditions was too narrow (Luman et al., 2008; Hüpen et al., 2020).

Overall, the measurement of SCRs has been shown to be a robust method to easily detect subconscious emotional arousal in anticipation and evaluation of DM. It yields a continuous measure that is related to activity in the sympathetic branch of the autonomic nervous system (Figner and Murphy, 2011). However, similar to many other indirect measurement methods, there are also some limitations. Subsequently, it must be taken into account that some participants can be “non-responders” (Figner and Murphy, 2011). In this study, we decided to include all measurements in the analyses as our statistical model corrected for individual differences in SCRs (Hüpen et al., 2019). Furthermore, there are contradictory recommendations on the pretreatment of the skin, whether the skin should be treated with water, oil or nothing at all before attaching the electrodes (Boucsein, 2012). We decided to follow the BIOPAC guidelines and used the saline paste supplied. Medication intake was also proven to affect activity of the autonomic nervous system (Bellato et al., 2020). Therefore, medication effect on behavioral results should be considered in future studies. Additionally, despite the potential individual lack of activity, there might also be environmental disturbances on the electrodermal recording that affect the signal. For instance, it was shown that electromagnetic noises such as overhead lights can disturb the signal and cause unrelated changes in the SCRs. We tried to avoid these artifacts by using low-pass filters and conducting the study in interference-free rooms, but a potential effect cannot be ruled out when considering the results. Furthermore, since the present study focuses on risky behavior in adults with ADHD it is important to consider that there may be limited comparability with child studies. Behavioral deficits in children may be deferred due to the delayed cortical maturation but may not persist in adulthood (Whelan and Mchugh, 2009; Dekkers et al., 2016; Koscielniak et al., 2016). In addition, a possible effect of comorbidities on the results must also be taken into account. For instance, patients with Antisocial Personality Disorder are also affected by deficits in affective functions (Glenn et al., 2013). Since the Mini-DIPS does not assess personality disorders, additional questionnaires should be included in future studies. Moreover, it should be taken into account that the present study investigated the specific functionality of emotional competence, but ADHD is particularly characterized by a heterogeneity of dysfunctions. However, the effectiveness of hot functions is still underrepresented in behavioral studies on ADHD. To gradually shed more light on this topic, future studies should specifically examine demographic effects such as age, gender, and education in relation to the ability of affective functioning in adult ADHD.

In conclusion, the present study is the first to investigate hot functions as underlying mechanisms for risky DM in adult ADHD. Results show significantly higher arousal before a DM and after feedback display in ADHD. However, ADHD participants were unable to use this physiological information to modify behavior. This finding is also confirmed by self-reports that showed a weaker ability in the perception of arousal and emotion in ADHD, whereas self-reported risk behavior was not altered compared to HC. Further research is needed to investigate how emotional processing can be influenced or improved in ADHD. However, our research underlines the importance of considering emotional therapeutic technics in the work with patients with ADHD.

## Data availability statement

The raw data supporting the conclusions of this article will be made available by the authors, without undue reservation.

## Ethics statement

The studies involving human participants were reviewed and approved by the Ethics Committee of the Medical Faculty of the University of Bonn. The patients/participants provided their written informed consent to participate in this study.

## Author contributions

EH, SL, and PH contributed to the conception and design of the study. EH, FK, and AH acquired the data and performed data analyses. PH designed the paradigm and performed data analyses. EH, MB, BH, CD, SL, and AP contributed to data interpretation and discussion of results. BA recruited patients with ADHD for study participation. EH wrote the first draft of the manuscript. All authors read and approved the submitted version.
